# Comparison of potential drug-drug interactions with metabolic syndrome medications detected by two databases

**DOI:** 10.1371/journal.pone.0225239

**Published:** 2019-11-14

**Authors:** Bovornpat Suriyapakorn, Pun Chairat, Suwanan Boonyoprakarn, Pimonwan Rojanarattanangkul, Wassana Pisetcheep, Natthaphon Hunsakunachai, Pornpun Vivithanaporn, Supakit Wongwiwatthananukit, Phisit Khemawoot

**Affiliations:** 1 Department of Pharmacy Practice, Faculty of Pharmaceutical Sciences, Chulalongkorn University, Bangkok, Thailand; 2 Osotsala the Community Pharmacy, Faculty of Pharmaceutical Sciences, Chulalongkorn University, Bangkok, Thailand; 3 Department of Pharmacology and Physiology, Faculty of Pharmaceutical Sciences, Chulalongkorn University, Bangkok, Thailand; 4 Department of Pharmacology, Faculty of Science, Mahidol University, Bangkok, Thailand; 5 Chakri Naruebodindra Medical Institute, Faculty of Medicine Ramathibodhi Hospital, Mahidol University, Samutprakarn, Thailand; 6 Department of Pharmacy Practice, Daniel K. Inouye College of Pharmacy, University of Hawai’i, Hilo, Hawaii, United States of America; 7 Department of Biochemistry and Microbiology, Faculty of Pharmaceutical Sciences, Chulalongkorn University, Bangkok, Thailand; 8 Preclinical Pharmacokinetics and Interspecies Scaling for Drug Development Research Unit, Chulalongkorn University, Bangkok, Thailand; Ehime University Graduate School of Medicine, JAPAN

## Abstract

**Background:**

Drug-drug interactions (DDIs) are one of the most common drug-related problems. Recently, electronic databases have drug interaction tools to search for potential DDIs, for example, Micromedex and Drugs.com. However, Micromedex and Drugs.com have different abilities in detecting potential DDIs, and this might cause misinformation to occur between patients and health care providers.

**Methods and findings:**

The aim of this study was to compare the ability of Micromedex and Drugs.com to detect potential DDIs with metabolic syndrome medications using the drug list from the U-central database, King Chulalongkorn Memorial Hospital in April 2019. There were 90 available drugs for the treatment of the metabolic syndrome and its associated complications, but six were not found in the Micromedex and Drugs.com databases; therefore, only 84 items were used in the present study. There were 1,285 potential DDI pairs found by the two databases. Micromedex reported DDIs of 724 pairs, while, Drugs.com reported 1,122 pairs. For the severity of the potential DDI reports, the same severity occurred between the two databases of 481 pairs (37.43%) and a different severity for 804 pairs (62.57%).

**Conclusion:**

Drugs.com had a higher sensitivity to detect potential DDIs by approximately 1.5-fold, but Micromedex supplied more informative documentation for the severity classification. Therefore, pharmacists should use at least two databases to evaluate potential DDIs and determine the appropriate drug regimens for physician communications and patient consultations.

## Introduction

Non-communicable diseases (NCDs) are a major health problem worldwide [1–3]. One of the major NCDs is metabolic syndrome, according to the NCEP ATP III definition, metabolic syndrome is present if three or more of the following five criteria are met: waist circumference over 40 inches (men) or 35 inches (women), blood pressure over 130/85 mmHg, fasting triglyceride level over 150 mg/dL, fasting high-density lipoprotein cholesterol level less than 40 mg/dL (men) or 50 mg/dL (women) and fasting blood sugar over 100 mg/dL [4, 5]. Metabolic syndrome is considered as a risk factor for various complications such as type 2 diabetes [6, 7]. The treatment of metabolic syndrome and its complications are usually related to multiple drug use, which might cause drug-drug interactions (DDIs) [8, 9]. DDIs can cause treatment failure, morbidity, and mortality to the affected patients [10, 11]. The severity of potential DDIs can be classified into contraindicated, major, moderate, minor, and none [12–13]. The severity levels of contraindicated and major seem to be a serious concern in drug dispensing in patients. In recent years, numerous tools have been developed to detect potential DDIs, and one of the most popular tools is online DDI databases; however, there are two major types of DDI databases, free online and copyrighted databases [14–16]. In the case of patient access for potential DDI determination, they usually use a free online database, e.g., Drugs.com. Meanwhile, health care providers usually detect potential DDIs using a copyrighted database, e.g., Micromedex. Ramos et al. reported that these two databases have different sensitivity and specificity in detecting potential DDIs between the prescriptions of HIV/AIDs patients in critical care [17]. Also, Bossaer et al. found that Drugs.com is the most sensitive DDI database for the detection of potential DDIs in oral antineoplastic combinations [18]. However, there are no studies regarding the ability of databases in detecting potential DDIs for the treatment of metabolic syndrome, which usually requires multiple drug use. The aim of this study was therefore to determine the different abilities of the two electronic databases in detecting potential DDIs with metabolic syndrome medications. The results of this study could increase the awareness of information obtained from different databases and lead to proper communication between metabolic syndrome patients and health care providers.

## Materials and methods

### Drug selection

**Fig 1:**
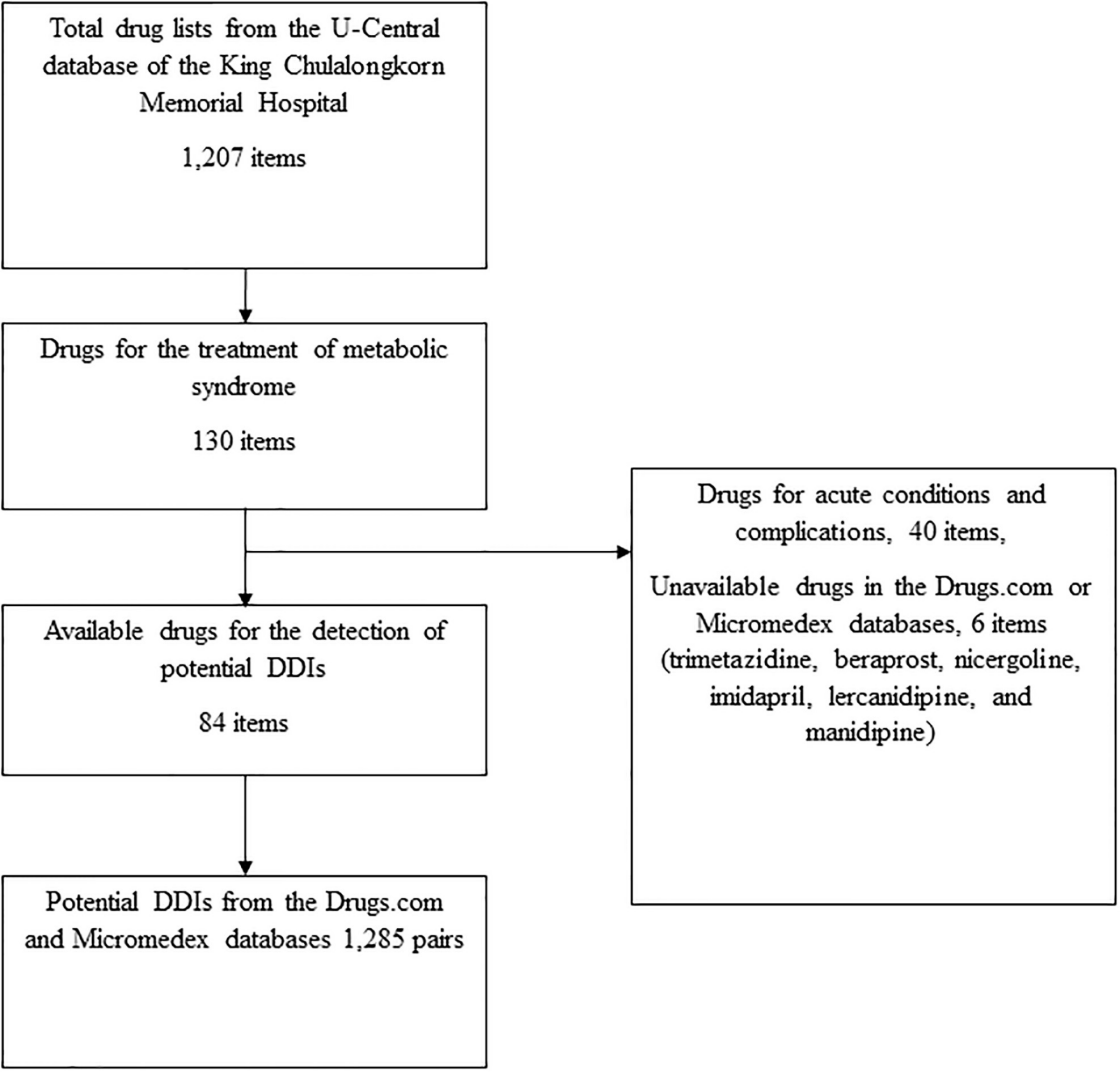
Flowchart of the study.

This descriptive study included a list of medicines for metabolic syndrome from the U-central database of the King Chulalongkorn Memorial Hospital that was taken on the 12^th^ April 2019 [19]. Of the 1,207 items in total, only 90 drugs were used for the treatment of the syndrome. Surprisingly, six drugs were not found in the Micromedex and Drugs.com databases; therefore, only 84 items were included in the study (Fig 1 and Table 1).

**Table 1:** Drug lists for the detection of potential DDIs.

Drug class	Drug groups	Drug lists
Cardiac drugs	Beta blockers	1. Atenolol 2. Bisoprolol 3. Carvedilol 4. Esmolol 5. Metoprolol 6. Nebivolol 7. Propranolol
	Antianginal agents	8. Ranolazine 9. Trimetazidine
Vasodilating agents	Phosphodiesterase inhibitors	10. Dipyridamole
	Arteriolar dilators	11. Hydralazine 12. Minoxidil
	Prostacyclin analogues	13. Iloprost 14. Beraprost
	Nitrates	15. Isosorbide dinitrate 16. Isosorbide mononitrate 17. Nitroglycerin
	Phosphodiesterase-5 inhibitors	18. Sildenafil
Peripheral vasodilators and related agents	Peripheral vasodilator agents	19. Nicergoline
	Xanthine derivatives	20. Pentoxifylline
Antiplatelets	Cyclooxygenase inhibitors	21. Acetylsalicylic acid
	Phospholipase-3 inhibitors	22. Cilostazol 23. Omega-3-acid ethyl ester
Antilipemic agents	Chelating agents	24. Cholestyramine
	Fibrates	25. Fenofibrate 26. Gemfibrozil
	HMG-CoA reductase inhibitors	27. Atorvastatin 28. Pitavastatin 29. Pravastatin 30. Rosuvastatin 31. Simvastatin
	Nicotinic acid	32. Nicotinic acid
	Selective cholesterol absorption inhibitors	33. Ezetimibe
	PCSK9 inhibitors	34. Evolocumab
Antihypertensive drugs	Angiotensin converting enzyme inhibitors	35. Captopril 36. Enalapril 37. Imidapril 38. Perindopril 39. Quinapril 40. Ramipril
	Angiotensin receptor blockers	41. Azilsartan 42. Candesartan 43. Irbesartan 44. Losartan 45. Olmesartan 46. Telmisartan 47. Valsartan
	Neprilysin inhibitors	48. Sacubitril valsartan sodium salt complex
	Thiazide and related diuretics	49. Hydrochlorothiazide 50. Indapamide 51. Chlorthalodine
	Alpha-2 adrenergic receptors	52. Methyldopa 53. Clonidine
	Alpha adrenergic antagonists	54. Doxazosin 55. Prazosin
	Calcium channel blockers	56. Amlodipine 57. Diltiazem 58. Felodipine 59. Lercanidipine 60. Manidipine 61. Nifedipine 62. Nimodipine 63. Verapamil
	Direct renin inhibitors	64. Aliskiren
	Endothelin-1 receptor antagonists	65. Bosentan 66. Macitentan
Diuretics	Diuretics loop diuretics	67. Furosemide
	Carbonic anhydrase inhibitors	68. Acetazolamide
	Osmotic diuretics	69. Mannitol 70. Glycerin
	Potassium-sparing diuretics	71. Amiloride 72. Spironolactone
Antidiabetic drugs	Alpha-glucosidase inhibitors	73. Acarbose
	Biguanides	74. Metformin
	Dipeptidyl peptidase-4 inhibitors	75. Linagliptin
	Glucagon-like peptide-1 receptor agonists	76. Liraglutide 77. Dulaglutide
	Meglitinide analogs	78. Repaglinide
	Sodium glucose co-transporter type 2 inhibitors	79. Dapagliflozin 80. Empagliflozin
	Sulfonylureas	81. Glimepiride 82. Glipizide
	Thiazolidinediones	83. Pioglitazone
	Insulins	84. Insulin

### Databases

IBM Micromedex Web Application Access was used in this study; it is a copyrighted database of IBM Corp., USA. The official registration and operations were conducted for academic purposes under the license from Chulalongkorn University. The DDI reports of the Micromedex database consisted of severity levels, documentation, onset, probable mechanism, management, literature, and references. The Drug Interactions Checker is a free online database provided by Drugs.com. This database is powered by four independent leading medical-information suppliers: Wolters Kluwer Health, American Society of Health-System Pharmacists, Cerner Multum, and Micromedex from Truven Health. The DDI reports of Drugs.com consisted of severity levels, management, probable mechanism, literature, and references.

### Identification of DDIs

The generic names of all the selected drugs were inputted into the database for potential DDI detection. All potential DDIs were recorded to determine the sensitivity and specificity of the reports. The severity of the potential DDIs from Micromedex was classified into five groups: contraindicated, major, moderate, minor, and none. Meanwhile, Drugs.com classified the potential DDIs into four groups: major, moderate, minor, and none. Micromedex classified the documentation of the outcomes as excellent, good, fair, and unknown, even though Drugs.com did not document these outcomes. Excellent documentation is defined as controlled studies have clearly established the existence of the interaction, good strongly suggests the interaction exists, but well-controlled studies are lacking, fair is defined as available documentation is poor, but pharmacologic considerations lead clinicians to suspect the interaction exists; or, documentation is good for a pharmacologically similar drug. Similarly, Micromedex also reported the onset of the potential DDIs as one topic; meanwhile, Drugs.com included the onset in the DDI monograph. All potential DDI reports were collected from the databases in April 2019.

### Data analysis

The data analysis was conducted using SPSS version 16 (SPSS Inc., US). The assessment of the agreement between the DDIs identified by the two databases was performed using the kappa index. A kappa value of 0.81–1.00 indicated an almost perfect agreement, 0.61–0.80 indicated a substantial agreement, 0.41–0.60 indicated a moderate agreement, 0.21–0.40 indicated a fair agreement, 0.00–0.20 indicated a slight agreement, and below 0.00 indicated a poor agreement [20].

## Results

From the 84 items analyzed, we found 1,285 pairs of potential DDIs from the two databases. Drugs.com reported DDIs of 1,122 pairs and Micromedex reported DDIs of 724 pairs. Of the 724 reported by Micromedex, the classification of severity was contraindicated in 23 pairs, major in 132 pairs, moderate in 566 pairs, and minor in 3 pairs. Drugs.com reported major DDIs of 130 pairs, moderate of 931 pairs, and minor of 61 pairs (Table 2). Among the 724 DDIs identified by the Micromedex, DDIs with excellent or good scientific documentation rating (47.79%) and stratifying the severity of DDIs according to documentation ratings are reported in Table 3.

**Table 2:** Comparison of the potential DDIs characterized by Micromedex and Drugs.com.

Severity	Micromedex n (%)	Drugs.com n (%)
Contraindicated	23 (3.18)	N/A
Major	132 (18.23)	130 (11.59)
Moderate	566 (78.18)	931 (82.98)
Minor	3 (0.41)	61 (5.43)
Total	724 (100.00)	1,122 (100.00)

N/A, not available

**Table 3:** Documentation of potential DDIs classified by Micromedex.

Severity	Excellent n (%)	Good n (%)	Fair n (%)
Contraindicated	5 (21.74)	13 (56.52)	5 (21.74)
Major	52 (39.39)	47 (35.61)	33 (25.00)
Moderate	8 (1.45)	219 (38.69)	339 (59.89)
Minor	0 (0.00)	2 (66.67)	1 (33.33)
Total	65 (8.98)	281 (38.81)	378 (52.21)

For the severity of potential the DDI reports, the same severity between the two databases of 481 pairs was 37.43%. The number of reports with a different severity were 804 pairs (62.57%) when comparing the two databases. Of these differing severity reports, major DDIs of 9 pairs reported by Drugs.com were not detected by Micromedex. On the contrary, Micromedex reported major DDIs for 21 pairs, although Drugs.com determined only minor to none DDIs as shown in Tables 4 and 5. The agreement between the severity reports of the two databases, as determined by the kappa value, was -0.055 (95% CI, -0.07068 to -0.03932, p < 0.001), which was considered to be a poor agreement between the two databases.

**Table 4:** The severity of the potential DDIs detected by Micromedex and Drugs.com.

Drug.com	Major n (%)	Moderate n (%)	Minor n (%)	None n (%)	Total n (%)
Micromedex					
**Contraindicated**	22 (1.71)	0 (0.00)	0 (0.00)	1 (0.08)	23 (1.79)
**Major**	83 (6.46)	29 (2.26)	3 (0.23)	17 (1.32)	132 (10.27)
**Moderate**	16 (1.25)	397 (30.89)	9 (0.70)	144 (11.21)	566 (44.05)
**Minor**	0 (0.00)	1 (0.08)	1 (0.08)	1 (0.08)	3 (0.24)
**None**	9 (0.70)	504 (39.22)	48 (3.73)	0 (0.00)	561 (43.65)
**Total**	130 (10.12)	931 (72.45)	61 (4.74)	163 (12.69)	1,285 (100.00)

**Table 5:** The significant difference of the severity in potential DDIs analyzed by Micromedex and Drugs.com.

Micromedex	Drugs.com	DDIs paired list with different severity
Contraindication	None	1. Aliskiren—Sacubitril
Major	Minor	1. Aspirin—Furosemide 2. Aspirin—Spironolactone 3. Nifedipine—Pioglitazone
Major	None	1. Acarbose—Aspirin 2. Acarbose—Glipizide 3. Acarbose—Pioglitazone 4. Aspirin—Amiloride 5. Aspirin—Chlorthalidone 6. Aspirin—Hydrochlorothiazide 7. Aspirin—Indapamide 8. Aspirin—Metformin 9. Clonidine—Metoprolol succinate 10. Clonidine—Metoprolol tartrate 11. Glimepiride—Pioglitazone 12. Glipizide—Pioglitazone 13. Metformin—Pioglitazone 14. Metoprolol succinate—Diltiazem 15. Metoprolol succinate—Verapamil 16. Metoprolol tartrate—Diltiazem 17. Metoprolol tartrate—Verapamil
None	Major	1. Amiloride—Azilsartan 2. Amiloride—Olmesartan 3. Amiloride—Spironolactone 4. Bosentan—Ranolazine 5. Clonidine—Metoprolol 6. Diltiazem—Metoprolol 7. Spironolactone—Azilsartan 8. Spironolactone—Olmesartan 9. Verapamil—Metoprolol

## Discussion

Drugs.com reported more potential DDIs than Micromedex by approximately 1.5-fold. These results were consistent with Ramos et al., who found that potential DDIs in HIV/AIDs patients detected by Drugs.com were more numerous than the Micromedex database indicated [17]. Bossaer et al. also mentioned that Drugs.com was more sensitive to detect the potential DDIs in cancer treatment than Micromedex [18]. This phenomenon may be explained by the fact that Drugs.com has a larger database which is contributed to by four suppliers, including Micromedex. Micromedex utilizes peer-review process to screen published medical studies as an evidence-based, pre-appraisal approach to assess the quality of documentation. The higher number of potential DDIs reported in Drugs.com might not always be fruitful in clinical practice. Some reports of potential DDIs are the combination of drugs used in the routine treatment of metabolic syndrome; for example, a combination of pioglitazone and metformin could generate benefits in controlling the symptoms of diabetes mellitus. The potential DDIs of these two antidiabetics seem to have positive clinical outcomes rather than negative adverse events. A large number of potential DDI alerts with limited documentation might lead to data alert fatigue to both health care providers and patients. Increased awareness regarding the discrepancy between the two databases should be made to both patients and providers who have access to these databases. Shared-decision making between providers and patients should be used for any significant potential DDIs in order to avoid alert fatigue and minimize liability.

Interestingly, we found that the two databases reported different severities in some potential DDI pairs. This major finding may cause drug-related problems in pharmacotherapy and may generate conflict between patients and health care providers. For example, Micromedex, which is preferred by health care providers, reported potential DDIs between aspirin and furosemide as being a major severity with excellent documentation. Meanwhile, Drugs.com, which is generally used by the patients, determined this interaction as being of minor severity. In contrast, Micromedex reported no potential DDI between amiloride and olmesartan; meanwhile, Drugs.com determined this potential DDI as a major event. In a case where patients develop severe cardiovascular complications while using these two agents together, health care professionals may be held liable for the harms a patient experiences. This is because Micromedex, which is usually utilized by health care providers, reports no concern for this DDI, but Drugs.com generally used by patients reported this as a major potential DDI. The evaluation of agreement between the more serious DDI reports of the two databases should be conducted and prioritized in order to prevent patient complications and also medical complaints.

We found that more than 80% of the potential DDIs of metabolic syndrome medications are pharmacodynamic interactions rather than those of pharmacokinetics. This phenomenon can be explained by the fact that metabolic syndrome is a complex pathological condition of the cardiovascular and endocrine systems. Combinations of several drugs for the treatment of metabolic syndrome are expected for the drug synergism of action, especially for pharmacodynamics rather than pharmacokinetic purposes. Interestingly, Drugs.com has a special feature to detect therapeutic duplications. This feature could reduce medication errors or the overdose of two drugs with an identical mechanism of action, e.g., atenolol and metoprolol. These two beta blockers have a similar mechanism of action by inhibiting the beta-1 receptors in the tissues of the heart. Even though this phenomenon seems prone to be caused by medication errors rather than true potential DDIs, it could be useful in some instances. In developing countries, patients can easily access dangerous medicines with a similar mechanism of action from drug stores or convenience stores. The determination of repetitive medicine with a similar mechanism of action by the free online database or mobile applications of Drugs.com might be useful in this circumstance.

The limitations of this study seem to be the dynamic changes of the drug list in the hospital, new drugs are entered and old drugs removed from time to time; therefore, this study made the cut off for the drug's list in early 2019. The current drug list in late 2019 might have had some changes from the time when we cut off the drug list. In addition, the two databases have frequently updated their potential DDI reports, so we collected all the potential DDI reports during early 2019. The current version of Micromedex and Drugs.com in late 2019 might report potential DDIs that are different from our study in early 2019. This study used only drugs from the electronic health record, thus not expanding the search to all drugs available in each of the respective classes. Therefore, there may be a greater (or lesser) discrepancy impact between the databases if all drugs in each class were included.

## Conclusion

The Drugs.com database had a higher sensitivity to detect potential DDIs, but Micromedex could provide more informative documentation for the severity classification. Both databases could be used to screen for potential DDIs, and the final justification should be conducted in accordance with the agreement and communication between patients and health care providers.

## Abbreviations

AIDS: Acquired Immunodeficiency Syndrome
DDIs: Drug-Drug Interactions
HIV: Human Immunodeficiency Virus
NCDs: Non-Communicable Diseases
NCEP ATP: the National Cholesterol Education Program of Adult Treatment Panel
